# Inflammatory Multiple-Sclerosis Plaques Generate Characteristic Metabolic Profiles in Cerebrospinal Fluid

**DOI:** 10.1371/journal.pone.0000595

**Published:** 2007-07-04

**Authors:** Norbert W. Lutz, Angèle Viola, Irina Malikova, Sylviane Confort-Gouny, Bertrand Audoin, Jean-Philippe Ranjeva, Jean Pelletier, Patrick J. Cozzone

**Affiliations:** 1 Centre de Résonance Magnétique Biologique et Médicale, UMR CNRS 6612, Faculté de Médecine de la Timone, Université de la Méditerranée, Marseille, France; 2 Service de Neurologie, Hôpital de la Timone, Marseille, France; Centre de Recherche Public-Santé, Luxembourg

## Abstract

**Background:**

Multiple sclerosis (MS), an inflammatory disease of the central nervous system, manifests itself in numerous forms and stages. A number of brain metabolic alterations have been reported for MS patients vs. control subjects. However, metabolite profiles of cerebrospinal fluid (CSF) are not consistent among the published MS studies, most probably due to variations in the patient cohorts studied. We undertook the first investigation of highly homogeneous MS patient cohorts to determine characteristic effects of inflammatory MS plaques on the CSF metabolome, including only patients with clinically isolated syndrome (CIS) with or without inflammatory brain plaques, and controls.

**Methodology/Principal Findings:**

CSF obtained by lumbar puncture was analyzed by proton magnetic resonance spectroscopy. 27 metabolites were quantified. Differences between groups of control subjects (n = 10), CIS patients with (n = 21) and without (n = 12) inflammatory plaques were evaluated by univariate statistics and principal component analysis (PCA). Seven metabolites showed statistically significant inter-group differences (p<0.05). Interestingly, a significant increase in β-hydroxyisobutyrate (BHIB) was detected in CIS with vs. without active plaques, but not when comparing either CIS group with control subjects. Moreover, a significant correlation was found, for the first time, between CSF lactate concentration and the number of inflammatory MS brain plaques. In contrast, fructose concentrations were equally enhanced in CIS with or without active plaques. PCA based on all 27 metabolites yielded group-specific clusters.

**Conclusions/Significance:**

CSF metabolic profiles suggest a close link between MS plaque activity in CIS patients on the one hand and organic-acid metabolism on the other. Our detection of increased BHIB levels points to a hitherto unsuspected role for this compound in MS with active plaques, and serves as a basis for further investigation. The metabolic effects described in our study are crucial elements in the explanation of biochemical mechanisms involved in specific MS manifestations.

## Introduction

Multiple sclerosis (MS) is a remarkably heterogeneous disease of the central nervous system [1]. At present, numerous clinical and biomedical studies are being carried out to characterize MS in its various forms and stages, with the aim of elucidating the individual mechanisms underlying their development and, ultimately, exploring specific targets for effective treatment. Among other methods, non-invasive in vivo ^1^H magnetic resonance spectroscopy (MRS) of the brain, as well as high-resolution MRS of cerebrospinal fluid (CSF) have been employed to detect differences in metabolic profiles between MS patients and controls. In vivo ^1^H MRS of acute lesions revealed increased total MRS-visible choline and lactate levels that tended to return to normal values after several months [2]. Furthermore, transiently increased myo-inositol (myo-Ins) and lipid MRS signals have been observed in MS, followed by N-acetylaspartate decreases [2].

While in vivo biochemical analysis of the brain is limited to a small number of metabolites, high-resolution ^1^H MRS of biofluids enables one to quantitate a much larger range of biochemical compounds, and thus to embark on a more detailed analysis of metabolic mechanisms. We have previously reported moderately increased CSF levels of lactate, creatinine, fructose and an unidentified compound in MS vs. control patients, while phenylalanine appeared to be decreased [3]. Our lactate findings were in agreement with Simone et al. [4], but at variance with other reports showing reduced lactate levels in CSF of MS patients [5], or no lactate differences at all [6]. There are several potential reasons for these discrepancies, besides differences in sample handling. The possibility that confounding factors may in fact determine the outcome of metabolomic analyses has recently been acknowledged by a number of reports, including also studies unrelated to neurological disease [7]. Since MS is a highly heterogeneous disease, diverging data may simply reflect different MS forms. Metabolic differences between CSF in relapsing-remitting vs. primary-progressive MS have been systematically investigated and were found to be negligible [3]. However, other characteristics such as the stage of MS and the prevalence of inflammatory vs. non-inflammatory plaques are rarely described in detail in reports comparing CSF metabolic profiles of MS patients to those of controls. To obtain more instructive results, a comparative study under well controlled conditions was required.

Increased lactate/creatine ratios have been found in CSF of MS patients with inflammatory, Gd-DTPA (gadolinium diethylenetriaminepentaacetate) contrast-enhanced (active) plaques at brain MRI (magnetic resonance imaging) when compared to patients without active plaques [4]. Since lactate is considered a marker of acute inflammation, it was hypothesized that the increased lactate/creatine ratios may reflect an increased CSF content of lactate stemming from inflammatory plaques [4]. Indeed, the presence of lactate, along with increases in total MRS-visible choline and lipids, has been demonstrated in active MS plaques by in vivo MRS [8]. The purpose of the present study was to determine, for the first time, (i) specific metabolic differences between MS with and without inflammatory plaques by comparing absolute (molar) metabolite concentrations in CSF of a highly homogeneous patient cohort, and (ii) the influence of either MS manifestation on the metabolic profile of normal CSF. Excellent patient group homogeneity was achieved by exclusively analyzing, for the first time, CSF from patients at the first episode (clinically isolated syndrome, CIS), and prior to glucocorticoid or any other treatment. The role of gender and age as potential confounding factors was also investigated. A better understanding of the metabolisms underlying specific MS manifestations should help develop tailored MS treatment protocols.

## Results

### General characteristics of metabolite profiles

**Figure 1:**
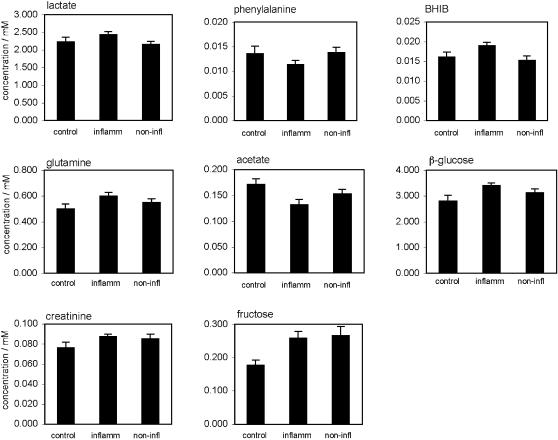
Selected CSF metabolite concentrations [mM] in patients with or without inflammatory brain MS plaques (abbreviated ‘inflamm’ and ‘non-infl’, respectively), and controls. Data represent means±s.e.m.

A total of 27 metabolites have been quantitated for CSF samples from 11 control patients, 21 CIS patients with active MS plaques (CIS group 1) and 12 CIS patients without active plaques (CIS group 2). Essential patient characteristics are given in Table 1. MRS signals used for quantitative evaluation were the most prominent proton resonances of valine, leucine, isoleucine, alanine, lactate, α-oxyisovalerate, α-hydroxyisovalerate, α-hydroxybutyrate, β-hydroxybutyrate, 1,2-propanediol, β-hydroxyisovalerate, β-hydroxyisobutyrate (BHIB), lysine, acetoacetate, acetate, creatine, creatinine, pyruvate, glutamine, citrate, glucose, fructose, myo-inositol, tyrosine, histidine, phenylalanine, formate, and three minor N-acetyl compounds. Mean values±standard errors of the means (s.e.m.) are given for concentrations of metabolites showing significant differences between groups (Figure 1). Significant differences are summarized in Table 2.

**Table 1:** Patient characteristics.

Patient ID	Sex	Age	number of active MS plaques in brain
1	F	32	1 CIS group 1
2	F	47	4
3	F	22	7
4	F	27	1
5	M	41	1
6	F	33	2
7	F	28	1
8	F	23	2
9	M	30	3
10	F	35	1
11	F	21	8
12	F	28	26
13	M	22	2
14	F	39	3
15	M	34	1
16	M	41	1
17	M	16	13
18	F	27	3
19	F	30	1
20	F	27	8
21	F	35	1
22	F	29	0 CIS group 2
23	F	28	0
24	M	39	0
25	F	32	0
26	F	48	0
27	F	42	0
28	M	24	0
29	F	32	0
30	F	23	0
31	F	25	0
32	F	49	0
33	M	33	0
34	F	36	0 controls
35	M	37	0
36	F	52	0
37	F	25	0
38	F	20	0
39	F	53	0
40	F	23	0
41	F	34	0
42	F	27	0
43	F	36	0

**Table 2:** Significant differences between individual CIS groups and controls.

	lac	phe	BHIB	gln	fru	ac	crn	β-glc
non-inflamm. vs. control					⇑			
inflamm. vs. control			(⇑)	⇑	⇑	⇓	⇑	⇑
inflamm. vs. non-inflamm.	⇑	(⇓)	⇑					
combined CIS vs. control				⇑	⇑	⇓		⇑

Upward (downward) arrows indicate significantly increased (decreased) CSF metabolite concentrations [mM] for CIS groups. The significance threshold was set to p<0.05. Parametric multiple-comparison tests (Fisher's PLSD) and nonparametric Mann-Whitney U tests yielded consistent results with respect to between-group significance (see Table 3). The arrow in parentheses indicates a trend close to significance (0.05<p<0.10). Means±s.e.m. are given in Figure 1. Abbreviations: lac, lactate; phe, phenylalanine; BHIB, β-hydroxyisobutyrate; gln, glutamine; fru, fructose; ac, acetate; crn, creatinine; β-glc, β-glucose.

**Figure 2:**
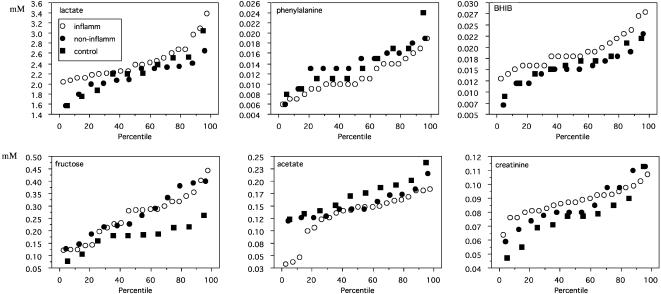
Percentile plots for selected CSF metabolite concentrations [mM] in patients with or without inflammatory brain MS plaques (abbreviated ‘inflamm’ and ‘non-inflamm’, respectively), and controls. These plots show the percentage of data less than or equal to an observed value. Marked deviations from normal distribution are readily recognized by major deviations from a straight line. The percentile plots for fructose illustrate the presence of CSF with particularly increased concentrations in CIS patients, independently of the presence of inflammatory plaques.

**Figure 3:**
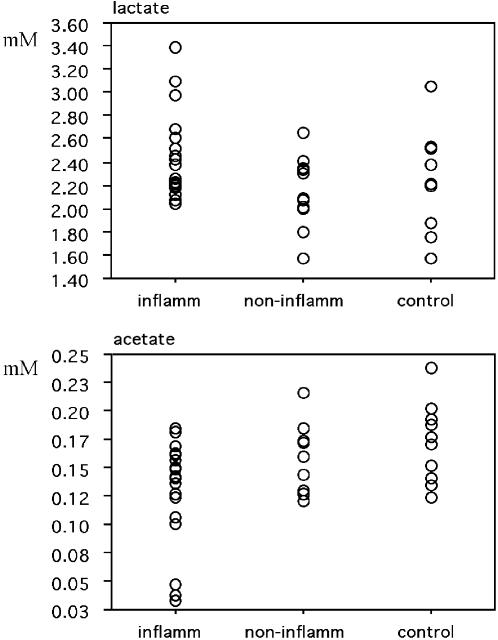
Scattergrams for lactate and acetate CSF concentrations [mM] in patients with or without inflammatory brain MS plaques (abbreviated ‘inflamm’ and ‘non-inflamm’, respectively), and controls. Marked deviations from normal distribution are readily recognized for lactate (inflamm) since data are clustered at low values (around 2.2 mM), but also for acetate (inflamm) where data are clustered at high values (around 0.16 mM).

CSF metabolite concentrations cover a broad range of values (Figures 2 and 3), with fructose varying between 0.10 and 0.45 mM (largest relative variation). Since the technical reproducibility of metabolite quantitation lies within a few percent for all but the least intense and/or strongly overlapping signals, the variation within a group is clearly dominated by the biological variability between subjects (standard deviations were about 15–20% of the means). For most metabolites, concentration ranges within the control group were equal to or somewhat larger than the corresponding ranges for the individual CIS groups. Fructose concentration ranges were an exception; they were considerably broader for the CIS groups than for controls (Figure 2). In fact, the differences in average fructose levels between either CIS group and controls are the result of an extension of the range of CIS fructose levels to higher values (>0.26 mM).

### Inflammatory plaques alter BHIB, lactate and phenylalanine CSF levels in CIS

**Figure 4:**
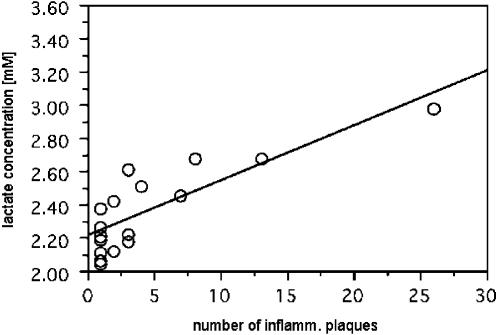
Lactate concentration [mM] in CSF of CIS patients with inflammatory plaques vs. number of inflammatory plaques, with linear fit.

Interestingly, β−hydroxyisobutyrate levels were significantly increased in CIS group 1 vs. CIS group 2 (+27%; Figure 1, and Tables 2 and 3). In addition, there was a trend (borderline significance) for BHIB increase in CIS group 1 vs. controls (Table 3). Moreover, lactate concentrations were moderately but significantly increased for CIS group 1 vs. CIS group 2 (+13%; Figure 1, and Tables 2 and 3), although the Kruskal-Wallis test did not suggest an overall inter-group difference for lactate (p = 0.154). To explain this apparent contradiction, we analyzed the data characteristics for CIS group 1 in more detail. Figure 3 shows considerable clustering of lactate concentrations around 2.2 mM for this group. Furthermore, Spearman rank analysis demonstrated a significant correlation between lactate levels and the number of inflammatory plaques (tied p = 0.0020, tied Rho = 0.691). A linear fit (Figure 4) resulted in a Y intercept (2.22±0.04 mM for a hypothetical number of zero active plaques) very close to the mean value found for CIS group 2 presenting no inflammatory plaques (2.16±0.08 mM) (Figure 1). Thus, (i) lactate concentrations in CIS group 1 cover a broad range of values depending on the number of active plaques, and (ii) the power of the Kruskal-Wallis test to detect lactate differences between groups is probably compromised by the data overlap between all three groups, in conjunction with major clustering at low lactate values in CIS group 1. No correlations were found between lactate concentrations in CSF and blood or urine (data not shown). For the decreased phenylalanine levels found in CIS group 1 vs. CIS group 2 (−21%, Figure 1), both the Mann-Whitney U test and Fisher's PLSD (Protected Least Significant Difference) test yielded a trend close to significance (Table 3).

**Table 3:** Significance of differences in CSF metabolites between CIS patients with and without inflammatory MS plaques and controls.

	Mann-Whitney U	Fisher's PLSD	compared groups
lactate	**0.0433**	**0.0356**	inflamm. vs. noninflamm.
BHIB	**0.0144**	**0.0127**	inflamm. vs. noninflamm.
BHIB	0.0851	0.0646	inflamm. vs. control
phenylalanine	0.0836	0.0932	inflamm. vs. noninflamm.
β-glucose	**0.0251**	**0.0069**	inflamm. vs. control
glutamine	**0.0384**	**0.0309**	inflamm. vs. control
creatinine	**0.0250**	**0.0403**	inflamm. vs. control
acetate	**0.0311**	**0.0123**	inflamm. vs. control
fructose	**0.0142**	**0.0179**	inflamm. vs. control
fructose	**0.0161**	**0.0203**	noninflamm. vs. control

P values for a nonparametric (Mann-Whitney U) and a parametric (Fisher's PLSD) test (see Materials and Methods for details of the statistical evaluation). Bold characters indicate significance at the p<0.05 level, while regular characters indicate borderline significance (0.05<p<0.10). Differences between CIS groups with (‘inflamm.’) and without (‘noninflamm.’) active MS brain plaques were analyzed based on the concentrations presented in Figure 1 (see also Table 2 for a summary).

### CSF metabolite levels in CIS groups vs. controls

Comparisons of CSF metabolite levels for CIS group 2 and controls yielded a statistically significant result solely for fructose which was increased by 50% in CIS group 2 (Tables 2 and 3). A somewhat smaller but equally significant fructose increase was observed for CIS group 1 vs. controls (+46%). Thus, virtually all of the CSF fructose increase in CIS patients vs. controls can be ascribed to metabolic changes that occur irrespectively of the presence of inflammatory MS plaques.

Differences between CIS group 1 and controls were significant for fructose, acetate, glucose, and, to a lesser degree, glutamine levels. These metabolites occurred at similar concentrations in CIS groups 1 and 2 (no significant differences). In fact, both CIS manifestations changed each of these metabolite levels in the same sense, compared to controls. However, CIS group 2 had smaller effects (p>0.05) than CIS group 1, except for fructose where effects were similar for both groups (Figure 1). Since both CIS groups 1 and 2 changed these metabolite levels in the same sense, significant differences were detected for fructose, acetate, glucose and glutamine when pooled CIS values were compared to controls (Table 2).

**Figure 5:**
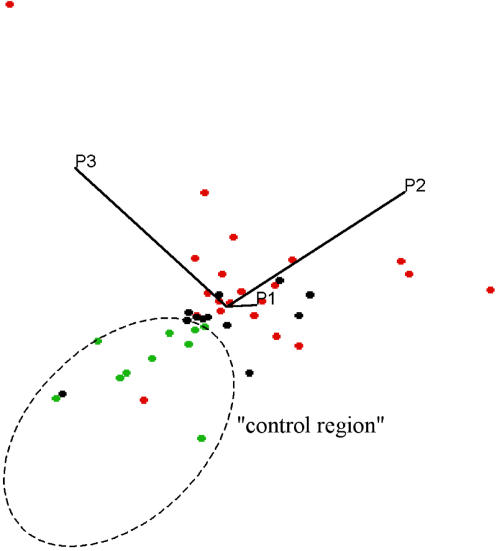
Two-dimensional projection of a three-dimensional CSF metabolite plot, based on the first three principal components obtained by PCA. All control subjects (green dots) are clustered within the broken ellipse that also contains one data point from each of the groups with and without inflammatory plaques (red and black dots, respectively).

**Figure 6:**
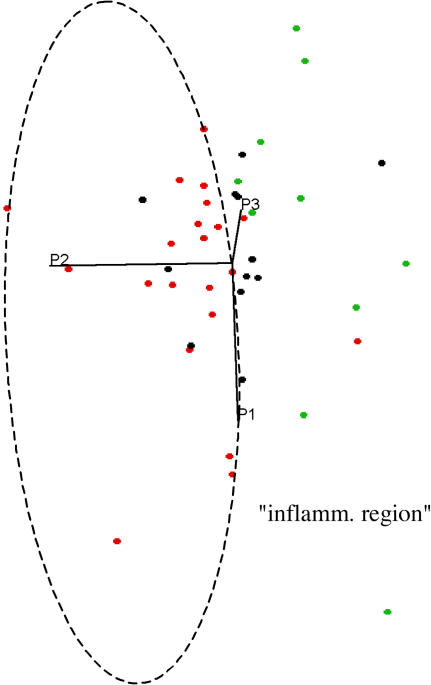
Two-dimensional projection of a three-dimensional CSF metabolite plot, based on the first three principal components obtained by PCA (projection angle changed relative to Figure 5). All CIS patients with inflammatory plaques (red dots), except for two, are clustered within the broken ellipse that also contains four data points from patients without inflammatory plaques (black dots).

Principal component analysis (PCA) unambiguously showed data clustering according to the three patient groups investigated. Note that our PCA examination is based on true, absolute metabolite concentrations rather than spectral points, and thus directly reflects actual metabolite levels (see Materials and Methods). Incomplete separation of data clouds, in particular for the two CIS groups (Figures 5 and 6), suggests that while each group is characterized by particular metabolic traits, these do overlap to some degree.

### Opposite trends for CIS with vs. without active plaques

As opposed to the metabolites described in the previous paragraph, no significant differences were detected for lactate and BHIB when both CIS patient groups were pooled together and compared to controls (Table 2). This is due to the fact that for lactate and BHIB, CIS groups 1 and 2 produced opposite trends (Figure 1): both metabolites were slightly reduced in CIS group 2 vs. controls (2.16±0.08 vs. 2.23±0.14 mM for lactate, and 0.015±0.001 vs. 0.016±0.001 mM for BHIB), but were increased for CIS group 1 (2.44±0.08 and 0.019±0.001 mM for lactate and BHIB, respectively). Consequently, these opposite trends compensated each other when both CIS groups were combined into one group. These opposite trends also explain why marked differences were detected only when CIS groups were compared to each other, but not when individual CIS groups were compared to controls.

### Correlations between metabolite levels and other parameters

Besides counting the number of inflammatory MS plaques which represent localized inflammation, we also determined non-local parameters characteristic of CNS inflammation (presence of oligoclonal IgG bands, and concentrations of oligoclonal and total IgG). However, no significant correlations between metabolite levels and immunoglobulin concentrations were found using the Spearman rank test. A Mann-Whitney U test resulted in significantly increased lactate levels for CSF samples from CIS patients demonstrating IgG oligoclonal bands vs. patients not showing oligoclonality, while no other metabolic differences were found between these two classes (data not shown).

The patients' age had no significant effects upon CSF metabolite levels, as verified by Spearman Rank correlation. There were no significant differences in metabolite concentrations between women and men, except for slightly enhanced glutamine (0.642±0.035 vs. 0.538±0.021) and creatinine (0.093±0.003 vs. 0.082±0.003) in men as evidenced by Mann-Whitney U tests (p = 0.0061 and 0.0058, respectively).

## Discussion

### Metabolic variations and patient homogeneity

All MS patients investigated in this study belong to a highly homogeneous population, in contrast to previously published ^1^H MR studies of CSF. For the first time, solely patients presenting the clinically isolated syndrome (CIS) were included, prior to any MS treatment. In addition, patients were carefully monitored to categorically exclude any possibility of interference by other (MS-unrelated) drug treatment. We found a number of metabolic CSF parameters differing between CIS patients with and without active plaques, and between either patient group and controls. These statistically significant differences are indicative of metabolic processes underlying and/or accompanying inflammatory MS plaques. However, CSF metabolite analysis cannot be recommended for diagnostic purposes since the metabolite concentration ranges for the three groups overlapped considerably.

### Increased β−hydroxyisobutyrate levels in CSF are closely associated with inflammatory MS plaques

One of the most striking results of our study is the first unambiguous detection of enhanced absolute (molar) BHIB and lactate concentrations in CSF of MS patients with vs. without inflammatory plaques. BHIB levels were positively correlated with the presence, but not with the number of inflammatory plaques. BHIB is a typical partial-degradation product of branched-chain amino acids (primarily valine) released from muscle for hepatic and renal gluconeogenesis [9]. The observed BHIB increase in CSF may in fact be due to an increased uptake (leakage?) from blood as discussed elsewhere [10], since BHIB is more concentrated in serum than in CSF [11], [12].

Notwithstanding, some of the BHIB increase may stem from astrocytes. Recent evidence suggests that these neural cells express moderate glucose 6-phosphate hydrolase and transporter activities [13], enabling gluconeogenesis and glucose release from the brain [13], [14]. Thus, increased levels of the gluconeogenesis substrate, BHIB, in CSF may be in part the result of a perturbation of astrocytic gluconeogenesis. The latter effect may be due to the presence of inflammatory plaques, or due to reduced cerebral blood flow as a result of a perturbation of microcirculation. Impaired microcirculation in MS may also explain lactate accumulation (see following section).

### Lactate levels in CSF are correlated with the number of inflammatory MS plaques

The statistically significant correlation between CSF lactate concentration and the number of inflammatory plaques found in our study (CIS group 1) clearly corroborates the assumption of a link between lactate production and inflammatory plaques [4]. This hypothesis is further supported by the finding that an extrapolation of the linear fit to zero inflammatory plaques yielded virtually the same lactate concentration as CIS group 2. In fact, lactate is a known marker of acute inflammation, although elevated lactate levels in CSF have also been found to reflect increased tissue acidosis and anaerobic glycolysis in other diseases [15]–[17]. It has been shown that in the presence of physiological stimulating factors, cultured macrophages derived from human monocytes synthesize increased amounts of lactate from glucose [18]. Thus, increased lactate production by immune cells is possibly at the origin of the increased CSF lactate levels observed in the presence of inflammatory MS plaques. Interestingly, lactate levels exhibit a stronger correlation with the number of inflammatory plaques than with markers of general CNS inflammation (IgG levels, oligoclonality). This suggests inflammatory plaques either directly produce lactate, or are closely linked to processes resulting in increased lactate production and/or release into CSF. Recent evidence suggests perturbed microcirculation in conjunction with hypoxia-like injury during MS plaque formation [19], [20]. This finding raises the possibility of increased lactate production by anaerobic glycolysis in and/or around emerging plaques, in an attempt to compensate for reduced oxidative ATP production. Some of the lactate produced in this way may be released into the CSF and explain increased levels for CIS patients with active plaques.

It is well established that the concentration of cerebral lactate directly depends on its rate of production in brain, while blood and CSF lactate concentrations are largely independent of each other [17], [21]. The lack of a correlation between lactate levels in CSF and serum of CIS patients, as determined in our study, confirms this finding. Note that CSF, although mainly produced in the choroid plexus (located in the fourth and lateral brain ventricles) and in the brain parenchyma, was sampled in the interspaces between lumbar vertebrae. Thus, processes occurring in the spinal canal, including also the spinal cord, may affect the results of CSF analyses, although the occurrence of active plaques in the spinal cord was not investigated. Another cause of lactate accumulation would be reduced clearance from CSF, but there is no evidence that changed lactate transport might play a significant role. Interestingly, oligoclonality, which is a marker of general CNS inflammation and present in 79–90% of MS patients, was less correlated with the number of inflammatory plaques than were lactate levels. Thus, as opposed to lactate, oligoclonality may be more indicative of CNS inflammation not directly linked to brain plaques (diffuse inflammation).

### MS heterogeneity and lactate changes

For our pooled MS group consisting of CIS patients with and without inflammatory plaques, no significant lactate increase was observed vs. the control group, owing to slightly decreased values for CIS group 2 (see Results). These findings suggest that the outcome of a comparison of lactate levels between MS patients and controls greatly depends on the composition of the MS cohort involved. Obviously, the proportion of patients with numerous active plaques plays a crucial role. However, other parameters may also be important, such as the proportion of CIS cases vs. cases with a history of multiple attacks, the proportion of treated vs. untreated patients, the characteristics of the control group chosen etc. Most certainly, CSF concentrations of metabolites other than lactate will also depend on the specific form and stage of MS under investigation.

### Glucose CSF concentrations are increased in CIS patients without active plaques

In contrast to lactate levels, glucose and fructose concentrations in CSF are generally determined by blood glucose concentrations [17]. The CSF concentration of glucose usually is about 60% of the blood concentration [17]. Normally, glucose enters CSF predominantly through facilitated diffusion, and follows blood level variations with a delay of approximately four hours [17]. A wide range of facilitating glucose transporters and isoforms have been identified in the CNS over the past years [22]. Increased glucose concentrations in CSF have been attributed to an increased permeability of the blood-brain barrier. Indeed, the CSF protein content is generally increased in MS patients vs. controls. This effect is commonly ascribed to a blood-brain barrier breakdown in a section of the brain or the spinal cord during MS attacks. Thus, the increased glucose levels found in CSF of MS patients with active plaques may partly stem from increased permeability of the blood-brain barrier. Since glucose is usually more concentrated in CSF than in brain tissue, CSF is thought to meet a part of the glucose demand of the brain by facilitated transport across the brain-CSF barrier [17].

### Fructose CSF concentrations are equally increased in CIS patients with and without active plaques

In general, fructose levels are considerable higher in CSF than in blood plasma [17]. While the mechanistic background of the fructose distribution in CSF vs. blood is unknown, it has been suggested that fructose is an end product of brain metabolism, accounting for the CSF:plasma fructose ratio greater than unity. In fact, a close correlation between glucose and fructose concentrations in CSF has been established [17], [23]. One glucose transporter (GLUT5) isoform has been found to functionally serve as a high-affinity fructose transporter [22]. The fact that fructose is characteristically enhanced in the CSF of MS patients irrespective of the presence of active plaques suggests enhanced transport across the brain-CSF barrier, while decreased fructose uptake by blood seems less likely.

### Glutamine and creatinine

Glutamine is an amino acid that plays an important role in brain metabolism. It is produced in large quantities and serves as a precursor of glutamate, the most abundant neurotransmitter in the CNS. However, it is also involved in numerous biosynthetic processes of the body, and is more concentrated in blood than in CSF. Due to substrate exchange between blood and CSF, the origin of increased glutamine in CSF of MS patients with inflammatory plaques vs. controls is difficult to assess. A recent in vivo ^1^H MRS study suggested increased glutamate, but not glutamine concentration in brain of MS patients [24]. Furthermore, our statistical evaluation showed that the CSF levels of glutamine and creatinine, another compound involved in nitrogen metabolism (e.g. in muscle protein degradation), varied significantly between the male and female patient subpopulations. Thus, the increased glutamine and creatinine levels found for CIS group 1 vs. controls may in part reflect the higher proportion of males in the former (29 vs. 10%). CIS group 2 consisted of 25% males, and their CSF glutamine and creatinine concentrations ranged between those of controls and CIS group 1. Further metabolic studies should be able to shed more light on the mechanisms of changed glutamine and creatinine metabolism in MS.

#### Phenylalanine

Phenylalanine CSF concentrations were slightly decreased in patients with active vs. inactive plaques (borderline significance, Tables 2 and 3). This aromatic amino acid is a precursor of the synthesis of the catecholamines, dopamine, noradrenaline and adrenaline [25]. Although phenylalanine can cause brain damage when present above a critical concentration, e.g. in phenylketonuria, it has been used, in combination with other compounds, as an effective drug to relieve MS symptoms. Its mechanism of action is thought to be associated with an increased synaptic availability of the neurotransmitter, noradrenaline, which may be depleted in MS patients [25]. Our results suggest that minor phenylalanine reduction in MS patients may be specifically linked to the presence of inflammatory plaques. However, further studies need to be carried out to test this hypothesis.

#### Acetate

Acetate is a metabolite of N-acetylaspartate and the neurotransmitter, acetylcholine, but is also involved, as acetyl-CoA, in lipid and carbohydrate metabolism (entry into the citric acid cycle). Some of us have previously reported that an accumulation of long-chain acyl CoA could result in the inhibition of acetyl CoA carboxylase, thereby diverting acetyl CoA from fatty acid synthesis to acetate formation via hydrolysis by acetyl CoA hydrolase (EC 3.1.2.2) [26]. In MS, acetate accumulation in brain has been interpreted in terms of reduced acetate incorporation into lipids, and the latter was suggested to play a role in altered myelin stability seen in MS [27]. However, the acetate decrease observed in the CSF of CIS group 1 and, to a lesser extent, CIS group 2, does not reflect these mechanisms. The biochemical pathways of acetate metabolism in MS await further elucidation.

### Conclusion

In clinically isolated syndrome (CIS), significantly increased β−hydroxyisobutyrate and lactate levels were detected in CSF of patients with vs. without active MS plaques. However, no significant differences were detected for these metabolites when comparing controls and CIS with or without active plaques. This finding underlines the heterogeneity of different manifestations of MS with respect to organic-acid metabolism. A compelling correlation was found between lactate concentration and the number of inflammatory plaques, suggesting a close link between plaque activity and lactate production. Several other metabolites (glucose, glutamine, acetate and creatinine) were found to vary significantly between controls and the group with active plaques only. These results contribute important elements to the investigation of metabolic processes involved in inflammatory MS plaques, and serve to guide further mechanistic studies of specific MS manifestations.

## Materials and Methods

### Patients

All patients gave their written informed consent to participate in this research protocol promoted by the French National Center of Scientific Research (CNRS) before entry. This protocol had been approved by the local Ethics Committee Marseille 1 (February 21, 2002).

A group of 33 CIS (clinically isolated syndrome suggestive of MS) patients was recruited from the department of Neurology (Timone Hospital, Marseille, J. Pelletier). Inclusion criteria were: age 18 to 45 years; occurrence of a first presumed inflammatory demyelinating event of acute onset in the central nervous system involving the spinal cord, the hemisphere or the brain stem; no previous history of neurological symptoms suggestive of demyelination; exclusion of alternative diagnoses by extensive investigations; presence of oligoclonal banding on CSF analysis; presence, on the initial MRI performed at time of inclusion, of one or more brain (including optic nerve) or spinal lesions (initial MRI included contiguous T_1_-weighted images with and without gadolinium enhancement, T_2_-weighted images and FLAIR images); patient not included in any other clinical trial.

Twenty-one of the CIS patients investigated showed inflammatory (active) plaques at brain MRI, as indicated by Gd-DTPA contrast-enhancement. The number of inflammatory plaques generally ranged between 1 and 10, although in two patients, 13 and 26 inflammatory plaques were detected, respectively (ID numbers 17 and 12, Table 1). The control group consisted of 10 patients suspected of multiple sclerosis. The average age of controls, patients with (CIS group 1) and without (CIS group 2) inflammatory plaques, was 34±11, 30±8 and 34±9 years, respectively. The sex ratio F/M was 9/1 for the control group, 15/6 for CIS group 1, and 9/3 for CIS group 2. MS was diagnosed according to McDonald's criteria [28].

For ethical reasons, it was not possible to have lumbar puncture performed on healthy volunteers. The control group did not present any abnormality at routine CSF analysis (immunoglobulins, B and T cells, glucose and total protein levels). Only one control subject had medical drug treatment (not related to inflammation) at the time of lumbar puncture, but did not show any CSF metabolite levels outside the range of the other controls. In general, 1 to 3 ml CSF were available for MRS analysis; in 5 cases, 340 µl to 1 ml were available. Blood and urine samples were obtained at the time of CSF collection to test if differences in CSF lactate concentrations were paralleled by similar differences in serum and urine.

### Detection of intrathecal IgG

For all patients, paired samples of CSF and serum were collected under sterile conditions after lumbar and venous punctures, respectively. Samples were stored at −80°C until free light chain (FLC) assays. All assays were performed under identical conditions. For each CSF sample, white and red blood cells were counted by light microscopy. IgG and albumin concentrations were quantified by immunonephelometry using a Behring analyzer (BNII, Dade Behring, Deerfield, IL). An index of the blood-CSF barrier function was obtained by calculating a CSF/serum albumin ratio. The IgG index and the intrathecal synthesis of IgG (Reiber's formula) were calculated as previously described [29]. Oligoclonal IgG banding was detected in unconcentrated fresh CSF samples according to the commercially available immuno-peroxidase method [29], [30]. Immunofixation performs comparably to other analytical methods such as isoelectric focusing [31].

### MRS sample preparation

CSF samples were stored at −80°C following lumbar puncture. For MRS analysis, CSF was lyophilized as described previously [32], and the lyophilizate was redissolved in 620 µl D_2_O. Sixty microliters of a 15 mM solution of (sodium) 3-(trimethylsilyl)-2,2′,3,3′-tetradeuteropropionate (TSP-d_4_) in D_2_O were added to each sample for chemical shift referencing and quantitation, and pH was adjusted with DCl and NaOD solutions to approximately 7.0. Lyophilization allowed for sample concentration up to a factor of 4–5, depending on the initial CSF volume available. Urine and serum samples were also stored at −80°C. Urine was lyophilized before MRS, while serum was analyzed following ultrafiltration to remove proteins.

### MR spectroscopy

#### Data acquisition

^1^H NMR spectra at 400.1 MHz were obtained on a 9.4 T AM-400 WB spectrometer from Bruker (Wissembourg, France), using a quattro nucleus probe for 5-mm tubes (tunable to ^1^H, ^31^P, ^13^C and ^19^F NMR resonance frequencies). Samples were spun at a rate of 20 Hz during NMR measurements. A standard Bruker Eurotherm BVT-3200 variable temperature unit was employed to maintain a sample temperature of 28°C. Biofluids were examined using 5-mm Wilmad 528-PP tubes (Carlo Erba-SDS, Val de Reuil, France). ^1^H NMR spectra were acquired under conditions that ensured full nuclear relaxation in all metabolites and the reference compound (pulse repetition time TR = 47.6 s, pulse width (90°) PW = 12.1 µs). Spectra were acquired using AQ = 6.56 s (64 k), preceded by water proton presaturation for 6 s (power setting 0.001 W).

#### Data processing

Before metabolite quantification, MR signals were apodized (exponential multiplication using LB = 0.5 Hz), Fourier transformed, and phase and baseline corrected. To quantitate the particularly weak aromatic amino acid signals, apodization with LB = 1.5 Hz was chosen for better signal-to-noise ratios. Spectra were integrated using Bruker's MDCON deconvolution routine; a 80% Lorentzian/20% Gaussian lineshape was found to optimally fit most metabolite signals. Metabolite signals were quantitated as described before for CSF of MS patients, to allow comparison with published results [3], [6]. NMR signal assignments were based on chemical-shift values previously presented for ^1^H NMR spectra of CSF samples [3], [33], and on addition of original compounds to CSF (“spiking”). BHIB was identified as described in a separate report [10].

### MR imaging

All subjects were explored on a 1.5 T Magnetom Vision Plus imager (Siemens, Erlangen, Germany). The MRI protocol included localizer images, fast transverse SE proton density-weighted and T_2_-weighted images (TE_1_/TE_2_/TR = 15/85/2600 ms; 44 contiguous slices, thickness = 3 mm; flip angle = 90°; FOV = 240 mm; matrix = 256^2^). A T_1_-weighted SE sequence (TE/TR = 10/650 ms, 25 contiguous slices, thickness = 5 mm; FOV = 240 mm; matrix = 256^2^) was performed before and 5 minutes after injection of Gd-DOTA (Dotarem, Gerbet, 0.1 mM/kg). MR images were analyzed by consensus during a single session by two observers (J.P.R. and B.A.) who were blinded to the clinical findings. The gadolinium-enhanced T_1_-weighted images were analyzed for the number of enhancing lesions.

### Statistics

Statistical tests were performed using Statview 5.0.1 software (SAS, Cary, NC, USA). First, homogeneities of variances were examined using F-tests, yielding equal variances for all metabolites and patient groups compared (except for creatinine in controls vs. CIS group 1, p = 0.049). Percentile plots (Figure 2) and scatter plots (Figure 3) were used to assess probability distributions since commonly available normality tests have little power to tell whether or not small sample groups are characterized by normal distribution [34], [35]. Significant deviations from normal distribution were detected for several metabolite/patient group combinations (Figures 2 and 3). Because parametric tests such as analysis of variance and linear discriminant analysis are appropriate only where the conditions of both equal variances and normal distribution are met, we used nonparametric tests (Spearman rank analysis and Kruskal-Wallis/Mann-Whitney U tests) to ascertain significant (p<0.05) correlations and differences between groups, respectively. However, nonparametric methods such as the Mann-Whitney U test are known to occasionally result in an underestimation of significance in cases where the assumptions of parametric tests apply fully or almost completely [35]. Moreover, since the Mann-Whitney U test is not designed to test for significance in multiple comparisons, results based on this two-sample test were verified by employing a parametric multiple-comparison method, Fisher's PLSD test (a nonparametric test for pairwise multiple comparisons was unavailable). Consistent p values for parametric and nonparametric tests clearly support the validity of our results (Table 3).

Principal component analysis was employed to examine clustering of data according to patient groups (JMP, SAS, Cary, NC, USA), using the 27 metabolite levels that were reliably quantitated. Our PCA method, based on actual (absolute) metabolite concentrations, contrasts with an alternative approach currently used in metabolomic MRS. The latter relies on intensities of spectral points or, more commonly, of “bins” or “buckets” (sums of a number of adjacent spectral points). One crucial advantage of employing metabolite concentrations (based on fitted peak areas) is that pattern recognition is not hampered by intersample peak position and line width variation [36]. These changes are the consequence of matrix effects (pH, ionic strength, etc.). Although there are techniques to align spectra prior to analysis, a significant limitation to these approaches is that overlapping peaks are highly problematic [36]. In contrast, deconvolution of overlapping peaks for metabolite quantitation fully takes into account these variations. A further advantage of using metabolite levels for PCA is that each of the resulting principal components directly represents a well defined linear combination of metabolite concentrations. On the contrary, binning-based principal components reflect combinations of spectral regions. Many of these are likely to represent superimposed MRS signals from different metabolites, while the relative contributions of individual metabolites to each region remain unknown.
